# Effects of Non-Stoichiometry on the Ground State of the Frustrated System Li_0.8_Ni_0.6_Sb_0.4_O_2_

**DOI:** 10.3390/ma14226785

**Published:** 2021-11-10

**Authors:** Evgeniya Vavilova, Timur Salikhov, Margarita Iakovleva, Tatyana Vasilchikova, Elena Zvereva, Igor Shukaev, Vladimir Nalbandyan, Alexander Vasiliev

**Affiliations:** 1 Zavoisky Physical-Technical Institute, FRC Kazan Scientific Center of RAS, 420029 Kazan, Russia; jenia.vavilova@gmail.com (E.V.); tmsalikhov@gmail.com (T.S.); ymf.physics@gmail.com (M.I.); 2 3rd Physics Institute, University of Stuttgart, 70569 Stuttgart, Germany; 3 Faculty of Physics, Lomonosov Moscow State University, 119991 Moscow, Russia; t_vasilchikova@yahoo.com (T.V.); vasil@mig.phys.msu.ru (E.Z.); 4 Faculty of Chemistry, Southern Federal University, 344090 Rostov-on-Don, Russia; ishukaev@sfedu.ru (I.S.); vbn@sfedu.ru (V.N.); 5 Quantum Functional Materials Laboratory, National University of Science and Technology “MISiS”, 119049 Moscow, Russia

**Keywords:** honeycomb lattice, magnetic frustration, metal oxides

## Abstract

The non-stoichiometric system Li_0.8_Ni_0.6_Sb_0.4_O_2_ is a Li-deficient derivative of the zigzag honeycomb antiferromagnet Li_3_Ni_2_SbO_6_. Structural and magnetic properties of Li_0.8_Ni_0.6_Sb_0.4_O_2_ were studied by means of X-ray diffraction, magnetic susceptibility, specific heat, and nuclear magnetic resonance measurements. Powder X-ray diffraction data shows the formation of a new phase, which is Sb-enriched and Li-deficient with respect to the structurally honeycomb-ordered Li_3_Ni_2_SbO_6_. This structural modification manifests in a drastic change of the magnetic properties in comparison to the stoichiometric partner. Bulk static (dc) magnetic susceptibility measurements show an overall antiferromagnetic interaction (*Θ* = −4 K) between Ni^2+^ spins (*S* = 1), while dynamic (ac) susceptibility reveals a transition into a spin glass state at a freezing temperature *T*_SG_ ~ 8 K. These results were supported by the absence of the λ-anomaly in the specific heat *C*_p_(T) down to 2 K. Moreover, combination of the bulk static susceptibility, heat capacity and ^7^Li NMR studies indicates a complicated temperature transformation of the magnetic system. We observe a development of a cluster spin glass, where the Ising-like Ni^2+^ magnetic moments demonstrate a 2D correlated slow short-range dynamics already at 12 K, whereas the formation of 3D short range static ordered clusters occurs far below the spin-glass freezing temperature at T ~ 4 K as it can be seen from the ^7^Li NMR spectrum.

## 1. Introduction

A rich variety of chemical and physical properties of honeycomb systems due to their crystal structure and peculiarities of electronic bonds has attracted an active interest of researchers in recent years. A well-known example is graphene [1,2], which has shown its unique properties such as high electrical and thermal conductivity, strength, and hydrophobicity. The 4d and 5d systems [3] with specifically strong spin-orbit entanglement are known to host a plethora of emergent quantum phases, such as the Weyl semimetals and topological insulators, as well as Kitaev physics [4,5]. In 3d honeycomb systems, the spin-orbit coupling is typically weak; however, the combination of low-dimensional nature of the lattice, strong electronic correlations, and enhanced quantum fluctuations promote the exotic spin liquid states [6], Berezinskii-Kosterlitz-Thouless transition [7,8], and suppression of long-range magnetic order [9]. Moreover, even though the honeycomb lattice is not magnetically frustrated for nearest spins, the competition of exchange interaction may occur due to significant contribution of next-nearest and next-next-nearest exchange couplings. This might result in formation of non-trivial spin structures, such as zig-zag or stripe type [10]. The presence of disorder and structural defects in honeycomb lattice can significantly modify the ground state of the system, in particular, formation of magnetic clusters with a finite correlation length and finite magnetization at T > 0 is possible [11]. Combination of such structural defects with magnetic frustration can result in an elusive phenomenon called “order by disorder” [12], or, in the presence of large amount of randomness, in spin-glass state [13].

In layered frustrated magnets at intermediate doping levels when the number of defects is high enough to destroy the long-range homogeneity, but insufficient for the total destruction of the two dimensionality of the magnetic matrix and short-range correlations, spin glass is a quite puzzling phenomenon. First, the possibility of a spin-glass state with non-zero freezing temperature in quasi two-dimensional objects was long considered to be a controversial issue [14]. Second, a spin-glass phase induced by geometrical frustration and disorder can often behave like a quantum or classical spin liquid in various aspects [15,16]. Third, the temperature evolution of static phases and spin excitations in a wide region above and below the glass transition point is not typical for ordinary spin glass. In particular, spin dynamics possesses the 2D character and spins remain fluctuating deeply below the freezing temperature [15,17]. At the same time, the clusters with local magnetic ordering are observed experimentally [9,17,18,19] and predicted by Monte-Carlo calculations [20] at some conditions. Such a combination of the properties of 3D spin glasses, 2D AFM ordering, 2D fluctuating magnetic states in in layered frustrated magnets with triangle, honeycomb and kagome geometry is often referred to as an unconventional spin glass [17,19,21,22]. An understanding of the origins and conditions of such unconventional spin state and possible methods for tuning from it to spin liquid or Neel state with domains is an actual problem of frustrated magnetism both for fundamental knowledge and for future practical applications. At the same time, the number of experimental works devoted to a detailed study of such a state is relatively small. This motivated us to study a two-dimensional honeycomb S = 1 magnet with an intermediate, in the mentioned above sense, number of defects using a combination of bulk and local experimental techniques. This combination has proven to be effective for investigating unconventional spin glasses [17,23,24,25].

For this purpose, we have chosen the complex transition metal oxide compound Li_0.8_Ni_0.6_Sb_0.4_O_2_. It is a Li-deficient derivative of zig-zag antiferromagnet Li_3_Ni_2_SbO_6_, which has 2D structural order of magnetic Ni^2+^ (*S* = 1) ions on a honeycomb lattice with non-magnetic Sb in the middle, while honeycomb layers being separated by Li ions [26,27,28]. This layered structure promotes two-dimensional ionic mobility, which makes such compounds to be the promising cathode materials [29,30,31]. Indeed, in previous work [32] it was shown that onset of ion mobility in Li_0.8_Ni_0.6_Sb_0.4_O_2_ is observed at T > 250 K. As it was mentioned above, the presence of structural defects in honeycomb 3d oxides, including those caused by cation substitution, affect not only the ionic mobility, but also radically change the ground state of the magnetic system [31,33]. Present work is addressed to the comprehensive study of the structural modification induced by Li deficiency and its impact on magnetic properties and the ground state of Li_0.8_Ni_0.6_Sb_0.4_O_2_ system.

The paper is organized as follows: experimental techniques are summarized in Section 2, the results of structure characterization, heat capacity, ac and dc susceptibility and ^7^Li nuclear magnetic resonance (NMR) are presented in Section 3 and discussed in Section 4. Main conclusions of work are summarized in Section 5. Structural details are given in the Supplementary Materials.

## 2. Experimental

Ion-exchange preparation of the Li-deficient Li_0.8_Ni_0.6_Sb_0.4_O_2_ sample from its sodium counterpart was reported previously [32,34]. Solid-state syntheses were performed using reagent-grade antimonic acid, hydrous nickel oxide (both analyzed gravimetrically) and dried lithium carbonate. The weighed portions were mixed thoroughly, pressed, reacted at 1070 K and, after regrinding and pressing, at 1270 K for 3 h. All samples were pale-green—a definite sign of purely divalent nickel (Ni^2+^)—because even small admixtures of Ni^3+^ (<<1%) are known to result in dark coloration.

For the X-ray powder diffraction (XRD) phase analysis, ARL X’TRA diffractometer equipped with a solid state Si (Li) detector and copper K_α_ radiation was used.

Measurements of static and dynamic magnetic susceptibilities were performed by means of a Quantum Design Magnetic Property Measurement System (MPMS) XL-7 magnetometer from Quantum Design. The temperature dependence of the static magnetic susceptibility was obtained at the magnetic field of B = 0.7 T in the temperature range 2–350 K. The ac susceptibility was measured at 2 < T < 25 K at frequencies 5, 59, 279 and 498 Hz. The dc field was not applied.

The specific heat measurements were carried out by a relaxation method using a Physical Property Measurement System (PPMS) system by Quantum Design, USA. The plate-shaped sample was obtained by cold pressing of the polycrystalline powder. Data were collected at zero magnetic field in the temperature range 2–210 K and under applied fields of 3, 6 and 9 T in the temperature range 2–30 K.

Nuclear magnetic resonance experiments were carried out using Redstone solid-state pulse spectrometer by Tecmag and resistive 0–1.5 T magnet by Bruker at the frequency 12 MHz. The NMR signals were observed on ^7^Li nuclei with spin *I* = 3/2 and gyromagnetic ratio γ = 16.546 MHz/T. The NMR spectra were obtained at a fixed frequency by step-by-step sweeping the field and integrating the solid-echo signal at each field point. ^7^Li longitudinal relaxation rate T_1_^−1^ was measured using a stimulated echo pulse sequence and ^7^Li transverse relaxation rate T_2_^−1^ was obtained by measuring the solid-echo integral as a function of *τ*, where *τ* is time between two *π*/2 pulses. Both relaxation rates were measured on the maximum of spectral intensity at each temperature.

## 3. Results

### 3.1. Chemisrty and Phase Relations

**Figure 1:**
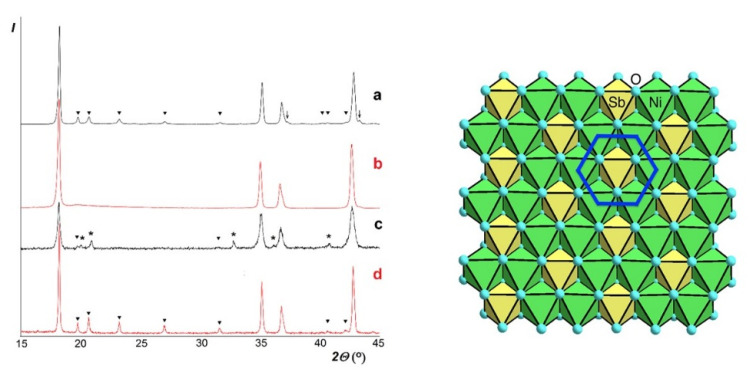
(**Left**) Comparison of low-angle parts of XRD patterns where most intensive foreign and superlattice reflections are observed. (a) Li_3_Ni_2_SbO_6_ [26]; (b) Li_0.8_Ni_0.6_Sb_0.4_O_2_ or “Li_2.4_Ni_1.8_Sb_1.2_O_6_”; (c) Same after 0.5 h at 1270 K; (d) Li_2.6_Ni_2.2_SbO_6_. Filled triangles (▼), superlattice reflections due to Ni/Sb ordering; asterisks (*), reflections from LiSbO_3_; arrows (↓), reflections from NiO (only found in a). (**Right**) Top view on the magnetic plane of Li_3_Ni_2_SbO_6_. The little light-blue circles are oxygen. Nickel ions are in the center of the green oxygen octahedra, antimony ions are in the yellow ones. The dark blue line marks the honeycomb motif in the magnetic structure of the plane.

The completely filled stoichiometric analogue, Li_3_Ni_2_SbO_6_ [26] showed distinct superlattice reflections characteristic of the honeycomb ordering of Ni and Sb (Figure 1a). Its pseudo-trigonal crystal structure was determined as monoclinic with lattice parameters listed in Table 1. In contrast, its lithium-deficient derivative, Li_0.8_Ni_0.6_Sb_0.4_O_2_ [32,34], only showed elevated background in place of superlattice peaks at 2*θ* ≈ 19–24° (Figure 1b). Therefore, its crystal structure was refined as truly trigonal (rhombohedral) with disordered Ni/Sb distribution over the same Wyckoff position. In addition, an 8% Li/Ni site inversion was detected, similar to Li_3_Cu_2_SbO_6_ [35] and opposite to Li_3_Ni_2_SbO_6_ [26]. However, other structural details, including lattice parameters, bond lengths and R-factors were not reported in our previous publication [32]. Therefore, they are reported here (see Supplementary Information, Figure S1 and Tables S1–S3).

Because of considerable difference in oxidation states and ionic radii between Ni^2+^ and Sb^5+^, we suppose that their local order should exist, as supported by the above-mentioned elevated background. Therefore, to facilitate comparison with the ordered counterpart, we recalculated its rhombohedral (hexagonal) lattice parameters to the monoclinic superlattice setting (Table 1).

The cation deficiency results in ca. 1% volume expansion. It is a typical feature of layered phases. However, it is usually only due to increase in the interlayer spacing whereas rigid octahedral layers contract because of decrease in average radius of octahedral cations: here, Sb^5+^ substituting for Ni^2+^. In the present case, however, the lattice expands in all directions. Supposedly, this is due to simultaneous Li^1+^ substitution for Ni^2+^. As mentioned previously [32], Li_0.8_Ni_0.6_Sb_0.4_O_2_ could not be prepared by direct high-temperature synthesis. Moreover, when prepared by the low-temperature ion exchange and heated at 1270 K for just 30 min, Li_0.8_Ni_0.6_Sb_0.4_O_2_ decomposed showing appearance of superlattice reflections and a second phase, LiSbO_3_ (Figure 1c). This superlattice phase is not Li_3_Ni_2_SbO_6_ since in this case there should be NiSb_2_O_6_, not found experimentally. Therefore, it was concluded that the decomposing reaction can be expressed by the following equation: 3Li_0.8_Ni_0.6_Sb_0.4_O_2_ = 0.4 LiSbO_3_ + 0.8 Li_2.5_Ni_2.25_SbO_6_. The composition with y = 0.2, Li_2.6_Ni_2.2_SbO_6_ synthesized by solid-state reactions at 1070 and 1270 K (Figure 1d), was found to be a single-phase superlattice similar to that obtained by calcination of Li_0.8_Ni_0.6_Sb_0.4_O_2_ (“Li_2.4_Ni_1.8_Sb_1.2_O_6_”) (Figure 1c). Its refined monoclinic parameters are: *a* = 5.1809 (4) Å, *b* = 8.9759 (3) Å, *c* = 5.1598 (4) Å, *β* = 109.580 (2)°, close to those of Li_3_Ni_2_SbO_6_ (see Table 1).

### 3.2. Magnetic Properties

**Figure 2:**
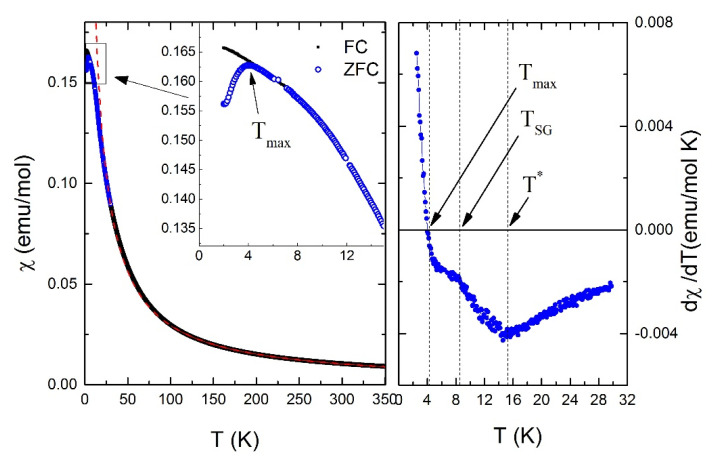
(**Left**) Static susceptibility of Li_2.4_Ni_1.8_Sb_1.2_O_6_ as a function of the temperature: field cooled (solid black circles) and zero field cooled (open blue circles) at external field of 0.7 T. Curie-Weiss fit is shown by red dashed curve. (**Right**) derivative of the low temperature part of zero field cooling static susceptibility.

The temperature-dependent bulk magnetic susceptibility *χ* of Li_2.4_Ni_1.8_Sb_1.2_O_6_ measured at B = 0.7 T is presented in Figure 2. As it was shown in previous work [32], the approximation of the high-temperature part of *χ* by Curie-Weiss law *χ* = *C*/(*T* − *Θ*) (*C* is a Curie constant) leads to the Weiss temperature *Θ* = −4 K. This temperature is comparable to stoichiometric compound Li_3_Ni_2_SbO_6_, where *θ* is 8 K and positive. This indicates that the magnetic interactions in the Li-deficient compound Li_2.4_Ni_1.8_Sb_1.2_O_6_ are predominantly of antiferromagnetic nature. Remarkably, the magnetic susceptibility *χ* deviates from the Curie-Weiss law at temperatures below 75 K, which indicates the onset of strong correlation effects. Moreover, in the low-temperature region, several features can be distinguished, where the curve changes its shape drastically (see d*χ*/d*T* in Figure 2, right panel): these temperatures are T* ≈ 12 K, *T*_SG_ ≈ 8 K and a wide maximum at *T*_max_ = 4 K. No sharp peak typical for magnetic transition to long-range AFM order is observed.

Notably, the bifurcation between the zero-field cooled (ZFC) and field-cooled (FC) branches appears below 8 K and becomes pronounced in the vicinity of 4 K. This divergence of FC and ZFC regimes is a characteristic hallmark of spin glasses—magnetically frustrated systems with a multidegenerate ground state [13]. In these systems, the spins order in a random, non-coplanar manner below the freezing temperature. In contrast to ferro- and antiferromagnets, spin glasses are characterized by the presence of short-range order but lack of long-range periodicity. The FC mode breaks the random distribution of the local magnetization direction and promotes freezing of spins in a predominantly in-field orientation, which leads to an increase in the bulk magnetization with decreasing temperature. In our experiment, the total magnetization continues to grow below the point of divergence even in the ZFC mode, showing a pronounced maximum only at half the freezing temperature that is unusual for classical spin glasses.

**Figure 3:**
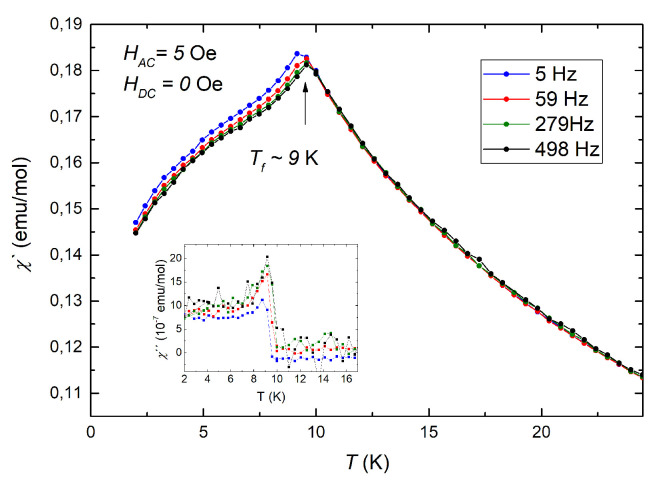
Temperature dependence of the real part of ac susceptibility, measured at different frequency with zero external dc magnetic field (main panel). Bottom inset shows temperature dependence of the imaginary part *χ*″ of ac susceptibility.

At the same time, it is well known that the magnetization in the freezing phase is strongly dependent on the time factor and the conditions for switching on a small external field required for measurements. These phenomena are the reason that a more informative and unambiguous method for studying spin glasses is to measure not the DC but the AC susceptibility. Therefore, to access a higher sensitivity to the magnetic transition, the ac susceptibility was measured at different frequencies with small driving field (*H*_ac_ = 5 Oe). The real part of the ac susceptibility *χ*′ (dispersion) exhibits a sharp cusp, as can be seen in Figure 3. At the same time, the imaginary part *χ*″ (absorption) associated with dissipative processes exhibits a step-like increase, which is typical for spin glass (lower inset in Figure 3) and excludes the case of the disordered AFM systems, where the value of *χ*″ is constant and remains zero below the transition temperature [36].

As expected for the spin glass systems, the position of maximum of *χ*′(*T*) which is called freezing temperature *T_f_* shifts to higher temperatures as observation frequency is increased. This shift can be described by different empirical models, namely critical scaling approach (i) and Vogel-Fulcher law (ii):

(1) $$\left(i\right)\;\tau =\tau_{0}\;{\left[\frac{T_{f}}{T_{SG}}-1\right]}^{-z\nu }\;\left(ii\right)\;\tau =\tau_{0}\exp\left[\frac{E_{A}}{k_{B}\left(T_{f}-T_{o}\right)}\right]$$

**Figure 4:**
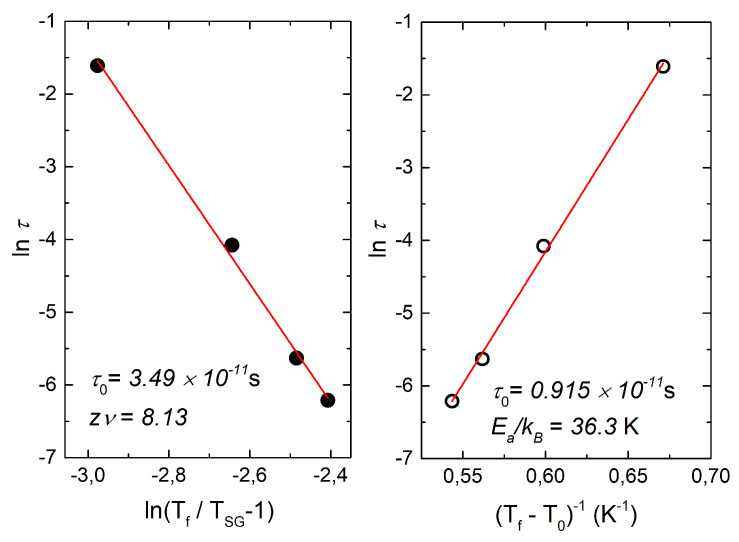
(**Left**) The fit of the shift of *T_f_* to the critical scaling and (**Right**) to the Vogel-Fulcher law.

Here, *k_B_* is the Boltzmann constant; the spin relaxation time *τ*_0_, the activation energy *E_A_*, the spin freezing temperature on static limit *T_SG_*, a characteristic temperature of inter-spin or inter-cluster interactions *T*_0_ and dynamical critical exponent *zν* are fitting parameters. The freezing temperature *T_f_* and the relaxation time *τ* (inversed AC-frequency) are determined from the experiment. Both models fit the experimental data reasonably well (see Figure 4) with parameters listed in Table 2. Obtained spin correlation times are of the order of 10^−11^ s, this value is at the border of the range of magnitudes typical for the canonical spin glasses (10^−10^–10^−14^ s [37]) and cluster spin glass, which demonstrates slower collective spin dynamics.

The typical activation energy *E_A_*/*k_B_* ~ 2 *T*_0_ values found for a canonical spin-glass CuMn [38], ~1.25 *T*_0_ for a geometrically frustrated system CaBaFe_4_O_7_ [39], however for cluster systems *E_A_*/*k_B_* is quite large: ~12 *T*_0_ for Li-doped CaBaFe_4−x_Li_x_O_7_ (*x* = 0.4) [39], ~3.1 *T*_0_ for U_2_CuSi_3_ [40] and ~7 *T*_0_ for La_0.5_Sr_0.5_CoO_3_ [41]. In our case, *E_A_*/*k_B_* ~ 4 *T*_0_ and from this we can infer that for Li_2.4_Ni_1.8_Sb_1.2_O_6_ is close to being a cluster spin-glass.

The critical exponent *zν* value obtained for critical scaling is 8.13 ± 0.33, which also belongs to the range between 4 and 13 that is usually observed in spin glass materials [37]. Moreover, it is intimately close to the theoretically predicted value of 9.3 for 3D spin glass [42,43].

### 3.3. Specific Heat Study

**Figure 5:**
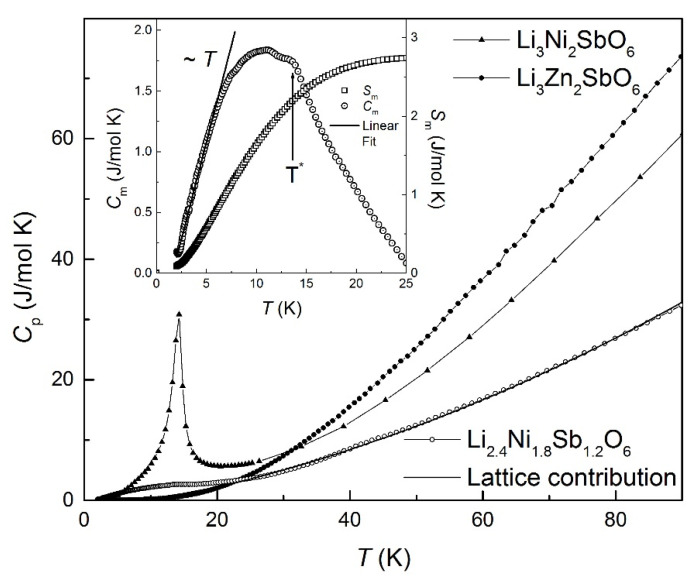
The temperature dependencies of specific heat *C*_p_(T) in zero magnetic field for Li_2.4_Ni_1.8_Sb_1.2_O_6_ (open symbols) in comparison with undoped Li_3_Ni_2_SbO_6_ (triangle symbols) and its isostructural non-magnetic analogue Li_3_Zn_2_SbO_6_ (filled symbols). The solid line represents the lattice contribution of the specific heat. Inset: the magnetic specific heat *C*_m_(T) (open circles) and the magnetic entropy *S*_m_(T) (open squares).

The temperature dependences of specific heat *C*_p_(*T*) of Li_3_Ni_2_SbO_6_ and Li_2.4_Ni_1.8_Sb_1.2_O_6_ systems are depicted in Figure 5. For undoped compound, in zero magnetic field, the *C*_p_(*T*) demonstrates a distinct *λ*-shaped anomaly at *T*_N_ ≈ 14 K, which is associated with Neel type 3D long-range magnetic order [26]. In contrast, in Li_2.4_Ni_1.8_Sb_1.2_O_6_ there is no apparent specific heat peak, but the broad maximum is observed in the region where magnetic susceptibility measurements reveal a spin-glass signature near 8 K. The lack of the *λ*-point in *C*_p_(*T*) is exactly one of the features for spin glass phase transition [13], owing to the much-released entropy above *T*_SG_.

The magnetic contribution to the specific heat *C*_m_(*T*) was determined by subtracting the lattice contribution using the data for the non-magnetic Li_3_Zn_2_SbO_6_. We assumed that this compound is reasonably isostructural to Li_2.4_Ni_1.8_Sb_1.2_O_6_ and can be used for an estimation of the pure lattice counterpart *C*_lat_(*T*) of the specific heat. Besides, we applied the standard scaling procedure [44] to the *C*_p_(*T*) data for Li_2.4_Ni_1.8_Sb_1.2_O_6_ and Li_3_Zn_2_SbO_6_.

The obtained magnetic part *C_m_*(*T*) (inset on Figure 5) demonstrates a rather broad hump, centered at *T* ≈ 10 K. Its amplitude, however, is significantly lower than Δ*C_theor_* = 29.9 J/mol K, expected from the mean field theory for *n* = 1.8 of Ni^2+^ (*S* = 1) ions [45]:

(2) $$\Delta C_{theor}=n\cdot 5R\;\frac{S\left(S+1\right)}{S^{2}+{\left(S+1\right)}^{2}}$$

In addition, one can see the slight shoulder at about T* = 14 K, which is satisfactory agrees with anomaly at susceptibility data (see Figure 2). The magnetic entropy S_m_(T) is also lower than the theoretical estimation Δ*S_theor_* = *nRln*(2*S* + 1) = 16.4 J/mol K [45], which confirms that most of the available entropy is already lost above *T_f_*. It is worth noting that, in Li_2.4_Ni_1.8_Sb_1.2_O_6_, low temperature dependence of Cm is linear and can be considered as a sign of spin glass behavior [28,46,47].

**Figure 6:**
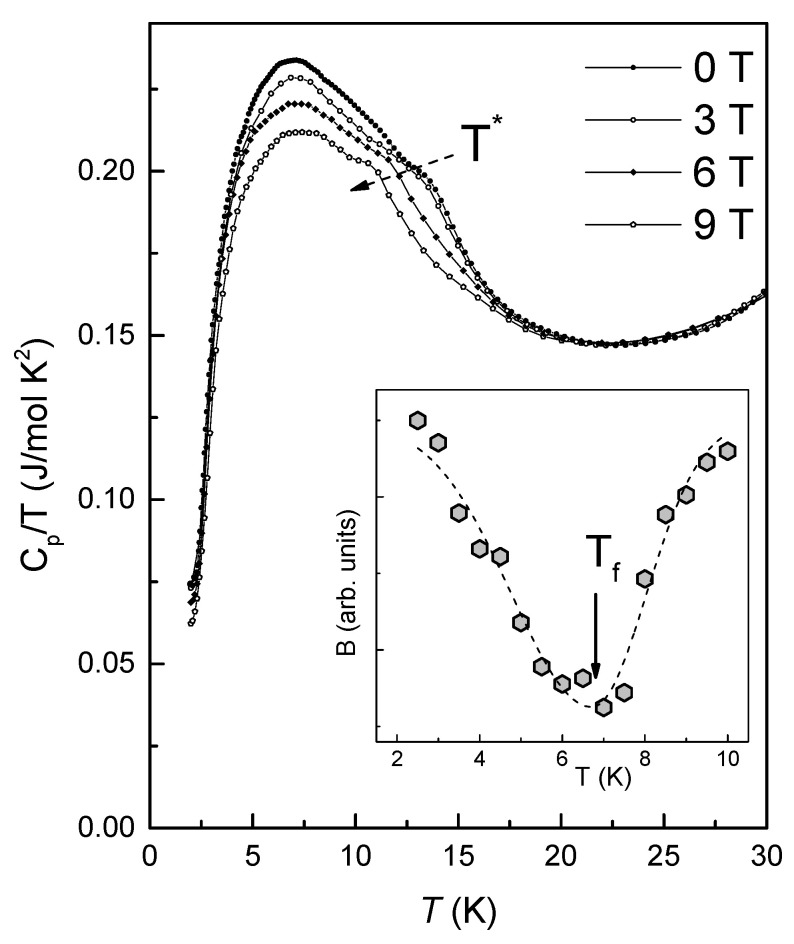
The temperature dependencies of specific heat *C_p_*/*T*(*T*) under various magnetic fields. The inset represents the temperature dependence of parameter B (see in text).

The temperature dependencies of specific heat *C_p_*(*T*) under external magnetic field clearly demonstrate the presence of the humps at T* (Figure 6). Moreover, this anomaly is field-sensitive, denoting to its antiferromagnetically ordering nature. The broad maximum on *C_p_*(*T*), located at around 7 K, probably can be attributed to the spin glass phase transition. For more accurate analysis of *T_f_* position we used approach, suggested by Brodale et al. [48]. From a sequential parabolic approximation *C_p_*(*H*) = *A* + *BH*^2^ of the specific heat in the temperature range 2 K to 10 K one can obtain the *T*-dependence of parameter *B* (inset in Figure 6). The found minimum at *T* ~ 7 K indicates this as the temperature of the spin-glass transition *T_SG_* of Li_2.4_Ni_1.8_Sb_1.2_O_6_.

### 3.4. Nuclear Magnetic Resonance

#### 3.4.1. ^7^Li NMR Spectra

The ^7^Li NMR studies were carried out in order to obtain information about local properties of the magnetic subsystem of the Li_2.4_Ni_1.8_Sb_1.2_O_6_ compound. Since the field and frequency dependence of the magnetic properties of spin glasses is strong, a relatively small external magnetic field of *B*_0_ = 0.724 T was applied. The results of high-temperature studies are presented in previous work [32], where the lithium mobility was investigated. Here we focus on the measuring of spectral and relaxation characteristics below room temperature.

**Figure 7:**
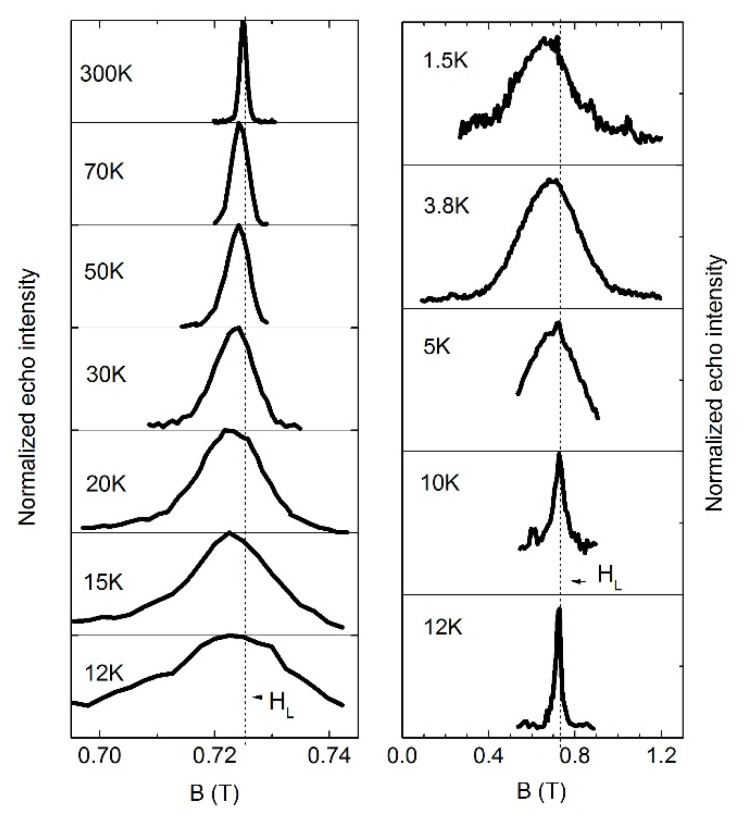
^7^Li NMR spectra at selected temperatures obtained at field of 0.724 T. Note that the scale on (**Right**) is enlarged compared to the (**Left**) one.

Figure 7 shows the temperature evolution of the ^7^Li NMR spectra. Despite the fact that lithium has a nuclear spin I = 3/2 and possesses quadrupole moment, the quadrupole satellites are not observed in the powder-averaged NMR line even at sufficiently high temperatures; therefore, the quadrupole splitting is less than the inhomogeneous linewidth. With decreasing temperature, the line gradually broadens, retaining the Gaussian-like shape, and the shape becomes more complex only at the lowest temperature.

**Figure 8:**
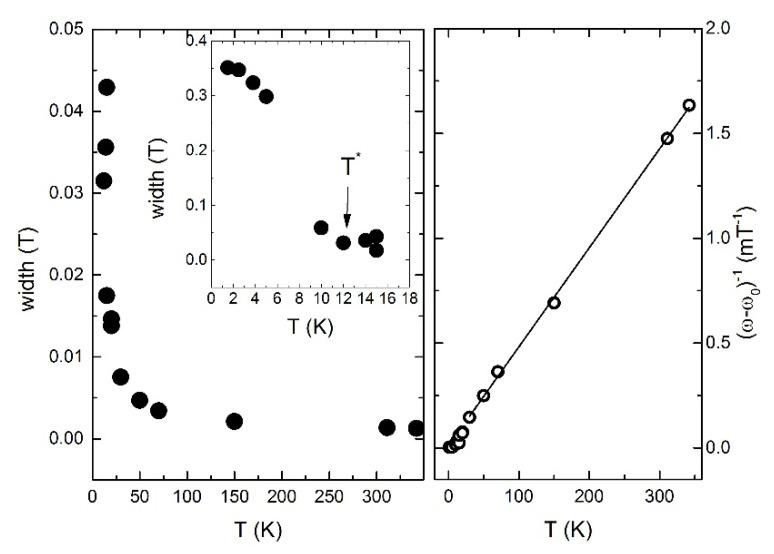
(**Left**) The temperature dependence of ^7^Li NMR linewidth ω(*T*). Inset presents the low-temperature part of the *ω*(*T*) dependence. (**Right**) Inverse linewidth vs. temperature (see text for details).

The temperature dependence of the full width at half maximum of peak is plotted in Figure 8. The linewidth *ω* is determined by the distribution of local magnetic fields and can be written as *ω* = *ω*_0_ + *ω*(*T*), where width on very high temperature limit *ω*_0_ = 0.67 mT. At *T* > 25 K the *T*-dependent part of the linewidth *ω*(*T*) follows a static bulk susceptibility and can be approximated by the Curie-Weiss law with *Θ* = −4.72 ± 0.06 K (solid line in Figure 8). Below 12 K, the linewidth sharply increases, indicating the emergence of an extremely strong distribution of local fields.

**Figure 9:**
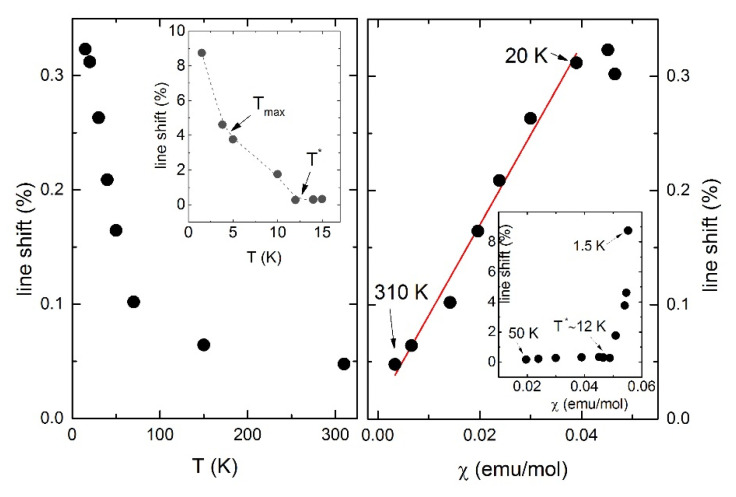
(**Left**) Temperature dependence of ^7^Li NMR line shift. Inset: the low-temperature part of the *K*(*T*), line is a guide for eye. (**Right**) local spin susceptibility versus bulk magnetic susceptibility. Inset shows low-temperature region.

The static part of the local susceptibility is represented by the shift of the NMR line *K*(*T*) = *K_sp_*(*T*) + *K*_0_, where *K_sp_* is the spin part of the shift, caused by hyperfine interactions between nuclear and electron spins. As is shown in Figure 9, *K_sp_* also follows the bulk of the susceptibility down to 20 K, strongly deviating upward with decreasing temperature. Two peculiarities can be noted in the temperature dependence of the local susceptibility below 20K. These are characterized by a change in the slope below *T* ≈ 10 K and *T* ≈ 4 K, and they are also observed in the bulk magnetic susceptibility curve.

#### 3.4.2. ^7^Li Nuclear Relaxation Rates *T*_1_^−1^ and *T*_2_^−1^

**Figure 10:**
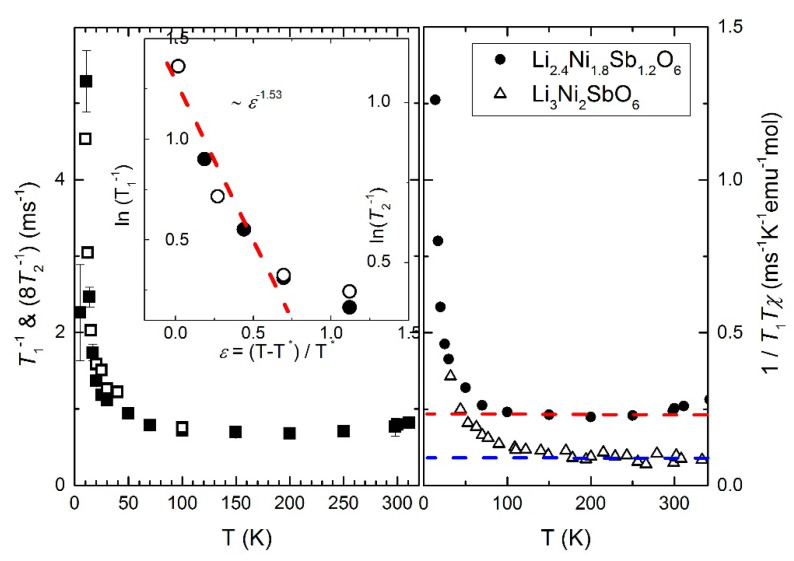
(**Left**) Temperature dependence of the ^7^Li spin-lattice relaxation rate *T*_1_^−1^ (solid squares) and transverse relaxation rate *T*_2_^−1^ (open squares) for Li_2.4_Ni_1.8_Sb_1.2_O_6_ at *B* = 0.724T. The inset shows a log-log plot of relaxation rate against the reduced temperature ε = |*T* − *T*_crit_ |/*T*_crit_ (*T*_crit_ = 11.7 K). The dashed line represents fit with the critical exponent according to Equation (9). (**Right**) 1/(*T*_1_*Tχ*) as a function of temperature for stoichiometric (open triangles) and lithium-deficient (solid circles) samples. Dashed lines show constant level for Li_2.4_Ni_1.8_Sb_1.2_O_6_ (red) and Li_3_Ni_2_SbO_6_ (blue).

Dynamic properties of the magnetic subsystem can be accessed by measurements of longitudinal and transverse relaxation rates *T*_1_^−1^ and *T*_2_^−1^. Due to the inability to resolve the quadrupole structure, the *T*_1_^−1^ was obtained by fitting the nuclear magnetization decay by a stretched exponential function. Details and an example of a fit can be found in previous work [32]. The spin-lattice relaxation rate *T*_1_^−1^ is almost temperature-independent at temperature range 70–300 K and increases rapidly below 70K (see Figure 10, below). The stretching parameter *b* deviates from 1 and approaches a value of 0.7 at the lowest temperatures [32]. The origin of such behavior is a distribution of *T*_1_ relaxation times, which directly indicates the inhomogeneity present in the system due to disorder effects.

The transverse relaxation rate *T*_2_^−1^, measured at T < 150 K follows the temperature dependence of longitudinal relaxation *T*_1_^−1^ (see Figure 10, below). Both times become critically small below 15 K, which leads to the wipe-out of NMR signal below 8 K. At these temperatures, due to the technical features of the method (*T*_2_^−1^ is comparable to the duration of the so-called hardware “ringing” of the measuring circuit and the “dead time” of the spectrometer) and physical reasons (the relaxation rate *T*_1_^−1^ becomes comparable with the frequency of magnetic resonance), correct measurement of relaxation rates and NMR spectra is difficult. The wipe-out temperature range is determined to be 4–15 K. Keeping these considerations in mind, one can indicate the observed sharp maximum of the temperature dependence of the relaxation rate in the vicinity of *T_SG_* ≈ 8 K.

## 4. Discussion

The temperature dependencies of the longitudinal *T*_1_^−1^ and transverse *T*_2_^−1^ relaxations of ^7^Li are shown in Figure 10. Spin-lattice relaxation in a concentrated magnet Li_2.4_Ni_1.8_Sb_1.2_O_6_ is mainly determined by the interaction of the nuclear spin with the electron spins of magnetic Ni^2+^ ions. It is almost constant in the middle temperature range, slightly increases at temperatures above 250 K due to the incipient activation motion of lithium ions [32] and sharply increases at low temperatures due to the growth of spin correlations and the slowing down of electronic fluctuations. These processes can be probed by the local dynamic susceptibility *χ*″ at the position of the nucleus, since:

(3) $$\frac{1}{T_{1}T}\propto \underset{q}{\sum }{\left|A_{⊥}\left(q\right)\right|}^{2}\frac{\chi^{\prime \prime }\left(q,\omega_{L}\right)}{\omega_{L}}$$

where $A_{⊥}\left(q\right)$ is the form factor, depending on wavevector ***q*** and $\omega_{L}$ is the Larmor frequency. From a comparison of the data for stoichiometric and nonstoichiometric compounds, it can be seen that spin correlations in Li_2.4_Ni_1.8_Sb_1.2_O_6_ begin to develop at temperatures below 70 K, which is much lower than for Li_3_Ni_2_SbO_6_. Moreover, in the middle temperature range, the value of the 1/*T*_1_*T* in the nonstoichiometric compound is larger, despite the fact that the hyperfine interaction constant with doping remains practically unchanged, as was established from measurements of the line shift. Apparently, the spectrum of electronic fluctuations in a sample with a lithium deficiency has a higher density in the low-frequency region, which makes relaxation more efficient.

The energy of the local spin fluctuations was estimated using formalism developed by Takeya et al. [17]. For spin *I* = 3/2 the relaxation rates *T*_1_^−1^ and *T*_2_^−1^ read as [49]:

(4) $$\frac{1}{T_{1}}\propto \gamma_{n}^{2}\left[\langle h_{x}^{2}\rangle +\langle h_{y}^{2}\rangle \right]\frac{\tau_{c}}{1+\omega_{L}^{2}\tau_{c}^{2}}$$

(5) $$\frac{1}{T_{2}}\propto \gamma_{n}^{2}\left(\frac{5}{2}\left[\langle h_{x}^{2}\rangle +\langle h_{y}^{2}\rangle \right]\frac{\tau_{c}}{1+\omega_{L}^{2}\tau_{c}^{2}}+\frac{1}{2}\langle h_{z}^{2}\rangle \tau_{c}\right)$$

where $\tau_{c}$ is a correlation time of fluctuations, $\langle h_{i}^{2}\rangle$ is a component of the fluctuating field on the nuclear position, *γ_n_* is a gyromagnetic ratio of the nuclei. Since *T*_1_^−1^ and *T*_2_^−1^ in the region 25 K < T < 150 K have the same temperature dependence (see Figure 10), the fluctuations of the local field are isotropic and *ωτ_c_* << 1. Then, at temperatures much higher than *T_SG_*, one can write:

(6) $$\frac{1}{T_{1}}\propto \frac{\gamma_{n}^{2}k_{B}T}{\mu_{B}^{2}}\;\chi \tau_{c}$$

and the characteristic energy of spin fluctuations

(7) $$\frac{\Gamma }{k_{B}}=\frac{\hbar }{\tau_{c}\left(T\right)k_{B}}\propto \frac{\hbar {\left(\gamma_{n}A_{hf}\right)}^{2}}{\mu_{B}^{2}}\;\chi T_{1}T$$

**Figure 11:**
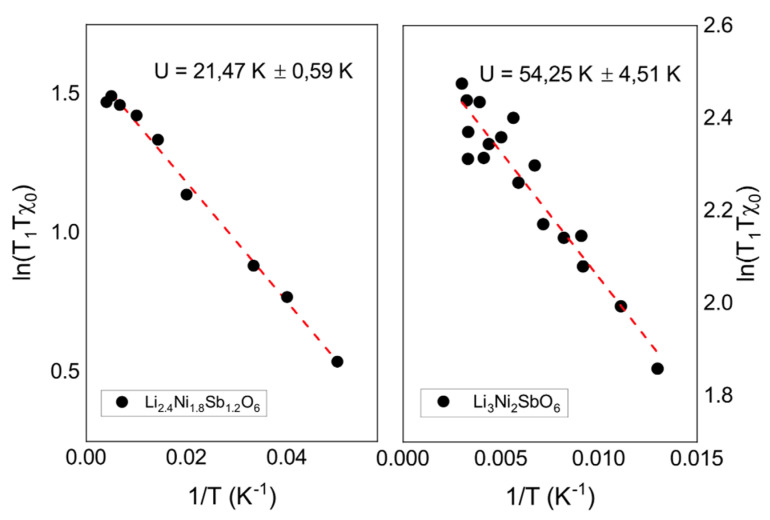
The ln(χ*T*_1_*T*) curve as a function of inverse temperature for (**Left**) lithium deficient and (**Right**) stoichiometric samples. Red dashed lines are fits according to Equation (8).

In this temperature range, the value of *χT*_1_*T* demonstrates an activation behavior:

(8) $$\chi T_{1}T\propto A\exp(-U/T),$$

where *U* = 21.47 ± 0.59 K corresponds to the local binding energy, i.e., with the magnitude of the spin exchange and the number of bonds (nearest neighbors) in the system of electron spins of nickel (Figure 11). Meanwhile, the value of binding energy in a stoichiometric sample, determined within the temperature range, where the local dynamic susceptibility does not deviate from the bulk static one, is significantly bigger and equal to *U* = 54.25 ± 4.51 K. Taking into account that the number of neighbors in the honeycomb lattice is three, the value of the exchange interaction is in good agreement with that obtained in thermodynamic studies [27]. Obviously, structural changes associated with the destruction of the hexagonal crystal lattice of Ni^2+^ ions in a lithium-deficient sample leads to a smaller number and a smaller value of bonds. As a result, electronic correlations are suppressed and the phase transition temperature decreases.

The critical behavior of the system at low temperatures can also be studied via nuclear relaxation processes. For this reason, the relaxation data was analyzed in similar fashion to the ac susceptibility. The temperature dependence of the relaxation rate in the critical regime slightly above *T_crit_* can be described by formula:

(9) $$T_{1}^{-1}={\left(T_{1}^{-1}\right)}_{0}+c\varepsilon^{-p}$$

Here, *ε* = |*T* − *T_crit_*|/*T_crit_* is reduced temperature, *c* is a prefactor, *T_crit_* is a critical temperature and p is a critical exponent (see Figure 10, insert to left panel). The obtained critical exponent value *p* = 1.67 is close to 2D Ising model (*p* = 1.5–1.9 [50,51,52]) and drastically different from the value obtained from the ac susceptibility. The obtained *T_crit_* = 11.7 K, which is close to T*. This gives evidence for the realization of the cluster spin glass in Li_2.4_Ni_1.8_Sb_1.2_O_6_, where the nuclear magnetic relaxation as a local technique characterizes the intracluster 2D critical spin dynamics, while from the AC susceptibility data we obtain the information about 3D glassy-like freezing of the bulk system. As mentioned above, further decreasing temperature leads to a dramatic increase in nuclear relaxation rates and, as a result, significant loss of NMR signal intensity. This wipe-out of the signal in unusually wide temperature range not only complicates the correct measurement of relaxation rates and spectra, but indicates an extended critical temperature range of 4–15 K, which contains a whole cascade of phase transformations of the magnetic system that freezes according to the cluster spin glass scheme. This is reflected primarily in the nonmonotonic temperature dependence of the static magnetic susceptibility and non-trivial behavior of the specific heat, but it also manifests itself in an unexpected way in local properties, in particular in NMR spectra at very low temperatures.

**Figure 12:**
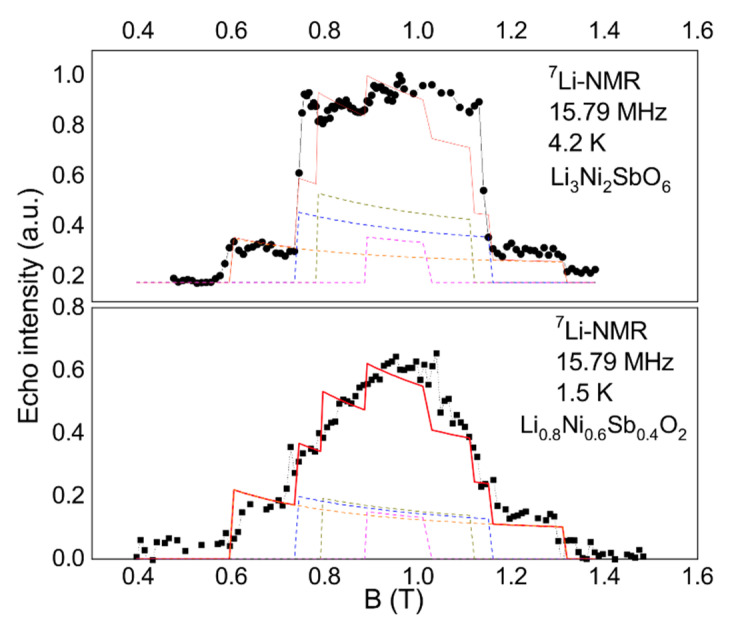
(**Upper**) The ^7^Li spectrum for Li_3_Ni_2_SbO_6_ at 4.2 K and (**Bottom**) for Li_2.4_Ni_1.8_Sb_1.2_O_6_ at 1.5 K obtained at frequency of 15.79 MHz. The red line is the result of dipole-dipole calculations; dashed lines represent the calculated contribution of different magnetically nonequivalent ^7^Li positions in frame of the powder averaging of the zig-zag model.

Below the *T_max_* = 4 K, the spectrum shifts sharply and its shape changes, ceasing to be strictly Gaussian. Some rectangular components of the spectrum can be distinguished, similar to those observed in the stoichiometric compound Li_3_Ni_2_SbO_6_ [27]. The rectangular structure of the spectrum is specific for regions with 3D antiferromagnetic order, while the width of the trapezoid is determined by the internal field magnitude at the position of the resonating nucleus in the powder sample. The modeling of internal fields carried out in Ref. [27] was based on the dipole model, taking into account the Ni^2+^ spins of the first coordination sphere of lithium nuclei. Our calculations demonstrated that it is possible to distinguish different types of ordered structures in a honeycomb sample by the number of magnetically nonequivalent lithium positions and the ratio of local fields on them (see Figure 12, upper panel). Moreover, the experimentally obtained ratio of the widths and intensities of the largest rectangular components unambiguously testified in favor of the zigzag structure. Interestingly, the ^7^Li NMR spectrum of Li_2.4_Ni_1.8_Sb_1.2_O_6_ obtained at 1.5 K also contains well-resolved rectangular components corresponding to at least two positions of Li with uniform local field corresponding to zig-zag AFM structure. At the same time, the main intensity of the spectrum comes from the central Gaussian-like part and the model does not fit to the experimental line shape there (see Figure 12, bottom panel). This can be interpreted as an emergence of the 3D short-range magnetically ordered clusters at *T_max_* = 4 K where the interplane exchange can bind the two-dimensional AFM-correlated regions. At the same time, most of the sample contains locally disordered spin configurations forming a spin glass pattern based on a quasi-triangular nickel lattice diluted with antimony ions.

Therefore, based on the results of complex studies, we assume the following scenario of the temperature transformation of the magnetic system of the layered Li_2.4_Ni_1.8_Sb_1.2_O_6_. With decreasing temperature, the rise of 2D antiferromagnetic correlation length leads to the appearance of AFM-correlated two-dimensional clusters in the regions of nickel-antimony planes with an undisturbed honeycomb structure. It manifests itself in the temperature dependence of the specific heat and static magnetization, as well as in the parameters of the NMR spectrum and the critical behavior of spin lattice relaxation at temperature T* = 12K which is close to T_N_ in a stoichiometric compound. A correlation length of several lattice constants and a rather long correlation time apparently characterizes the spin dynamics in such clusters. At the same time, the spins in the disturbed part of the nickel—antimony planes remain quasi-free and act as a gradually solidifying matrix separating the correlated clusters. 3D spin glass freezing of this matrix occurs at a temperature *T_SG_* = 8 K. The growth of interplane correlations due to the interplane exchange interaction *J_interplane_* << *J_intraplane_* connects 2D clusters in neighboring layers at temperatures below *T_SG_*. As a result, three-dimensional clusters with a local static order are formed at *T_max_* = 4 K whose magnetic structure is close to the zig-zag order of the parent compound. Although in some honeycomb systems with defects, clusters are transformed to long-range magnetic order at low temperatures (see for example, [9]), the ground state of more disordered magnetic system of Li_0.8_Ni_0.6_Sb_0.4_O_2_ is 3D cluster spin-glass. Despite this, the regions with a sufficiently large structural and magnetic correlation length persist in crystal matrix, leading to the formation of clusters with magnetic order.

## 5. Conclusions

Lithium-deficient Li_0.8_Ni_0.6_Sb_0.4_O_2_ crystal structure was refined as truly trigonal (rhombohedral) with disordered Ni/Sb distribution over the same Wyckoff position and 8% Li/Ni inversion. The new superlattice phase is Ni-enriched and Li-deficient (Li_3−2y_Ni_y_)Ni_2_SbO_6_. This intermediate level of the Li-Ni site inversion has led to a number of non-trivial magnetic properties. In the paramagnetic regime, modifications of the lattice parameters cause a change in the balance of the in-plane antiferromagnetic and ferromagnetic exchange integrals and lead to the antiferromagnetic sign of the biggest interaction. The transition into the long-range ordered AFM state is not observed in the Li_0.8_Ni_0.6_Sb_0.4_O_2_ compound, but a non-trivial cluster spin glass state is realized. It is characterized by a stepwise transformation of the magnetic system at low temperatures as a consequence of the balance between the growth of intra-cluster and inter-cluster correlations. It was found that, with decreasing temperature, the system of two-dimensional slow dynamic spin clusters undergoes the freezing transition. In this static state, the growth of the interplane correlation length promotes the formation of the static short-range ordered regions coexisting with a spin-glass state.

## Figures and Tables

**Table 1:** Crystal lattice parameters of Li_3_Ni_2_SbO_6_ and Li_0.8_Ni_0.6_Sb_0.4_O_2_.

Composition	Hexagonal Setting (*R*-3*m*)		Monoclinic Setting (*C*2/*m*)			
	*a*, Å	*c*, Å	*a*, Å	*b*, Å	*c*, Å	*β*, °
Li_3_Ni_2_SbO_6_ “LiNi_0.67_Sb_0.33_O_2_”	Pseudocell		True supercell			
	2.9897	14.5575	5.1828	8.9678	5.1578	109.69
Li_0.8_Ni_0.6_Sb_0.4_O_2_ “Li_2.4_Ni_1.8_Sb_1.2_O_6_”	True cell		Supposed supercell			
	3.0027	14.6103	5.2008	9.0080	5.1695	109.59

**Table 2:** Parameters obtained from the data in Figure 4 fitted with the critical scaling approach and Vogel-Fulcher law.

Parameter	Critical Scaling	Vogel-Fulcher
*τ*_0_[s]s	(3.49 ± 0.61) × 10^−11^	(1.92 ± 0.23) × 10^−11^
*E_A_*/*k_B_* [K]	-	36.3 ± 1.1
*T*_SG_ and *T*_0_ [K]	8.9	7.9
*zν*	8.13 ± 0.33	-

## Data Availability

Not applicable.

## Supplementary Materials

The following are available online at https://www.mdpi.com/article/10.3390/ma14226785/s1, Figure S1: Results of the Rietveld refinement: XRD profile and polyhedral view of the crystal structure of Li_0.8_Ni_0.6_Sb_0.4_O_2_. Table S1: Crystal structure refinement details. Table S2: Atomic positions. Table S3: Bond lengths and bond angles. References [53,54,55] are cited in the supplementary materials.
