# Supplementary material for: Effects of Non-Stoichiometry on the Ground State of the Frustrated System Li0.8Ni0.6Sb0.4O2
Source: Materials (Basel). 2021 Nov 10;14(22):6785. doi: 10.3390/ma14226785 (PMC8621701; doi:10.3390/ma14226785)
Supplement: Supplementary file 1 [file materials-14-06785-s001.zip › materials-1424539-supplementary.pdf]

materials

Supplementary Materials

Effects of Non-Stoichiometry on the Ground State of the Frustrated System Li0.8Ni0.6Sb0.4O2

Evgeniya Vavilova [1], Timur Salikhov [1], Margarita Iakovleva [1,2], Tatyana Vasilchikova [3], Elena Zvereva [3,†], Igor Shukaev [4], Vladimir Nalbandyan [4] and Alexander Vasiliev [3,5,*]

1 Zavoisky Physical-Technical Institute, FRC Kazan Scientific Center of RAS, 420029 Kazan, Russia;
jenia.vavilova@gmail.com (E.V.); tmsalikhov@gmail.com (T.S.); ymf.physics@gmail.com (M.I.)
2 3rd Physics Institute, University of Stuttgart, 70569 Stuttgart, Germany
3 Faculty of Physics, Lomonosov Moscow State University, 119991 Moscow, Russia;
t_vasilchikova@yahoo.com (T.V.), vasil@mig.phys.msu.ru (E.Z.)
4 Faculty of Chemistry, Southern Federal University, 344090 Rostov-on-Don, Russia;
ishukaev@sfedu.ru (I.S.); vbn@sfedu.ru (V.N.)
5 Quantum Functional Materials Laboratory, National University of Science and Technology "MISiS", 119049 Moscow, Russia
* Correspondence: anvas2000@yahoo.com
† deceased.

Citation: Vavilova, E.; Salikhov, T.; Iakovleva, M.; Vasilchikova, T.; Zvereva, E.; Shukaev, I.; Nalbandyan, V.; Vasiliev, A. Effects of Non-Stoichiometry on the Ground State of the Frustrated System Li0.8Ni0.6Sb0.4O2. Materials 2021, 14, 6785. https://doi.org/10.3390/ma14226785.

Academic Editor: Dmitry A. Shulyatev

Received: 30 September 2021
Accepted: 7 November 2021
Published: 10 November 2021

Publisher's Note: MDPI stays neutral with regard to jurisdictional claims in published maps and institutional affiliations.

Copyright: © 2021 by the authors. Licensee MDPI, Basel, Switzerland. This article is an open access article distributed under the terms and conditions of the Creative Commons Attribution (CC BY) license (http://creativecommons.org/licenses/by/4.0/).

A. Experimental details

The powder diffraction pattern [1] for the structural refinement was taken in Cu K_α radiation using a rotating-anode Rigaku instrument equipped with a secondary-beam graphite monochromator. Structural analysis was performed by the Rietveld method implemented in GSAS+EXPGUI suite [2, 3].

B. Structural results

The crystal structure was successfully refined with parameters listed in Tables 1, 2, 3 and refinement results are shown in Fig. S1.

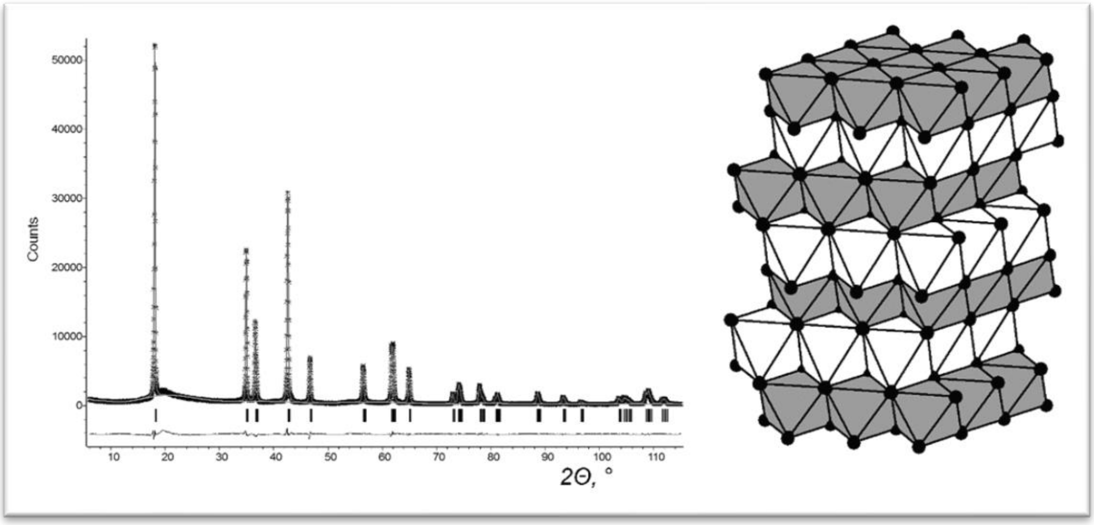

Figure S1. Left: Results of the Rietveld renement of $Li_{0.8}Ni_{0.6}Sb_{0.4}O_2$ XRD prole [1]: asterisks are experimental points; line represents calculated prole; line at the bottom is the difference plot; vertical bars are the Bragg positions. Right: Polyhedral view of the crystal structure [1], where $(Ni,Sb)O_6$ octahedra with small admixture of Li are shown in gray and LiO_6 octahedra, partially occupied with small admixture of Ni are shown in white. See Tables I,II, III for details.

Table S1. Crystal structure refinement details for $Li_{0.8}Ni_{0.6}Sb_{0.4}O_2$.

**Crystal system**		**trigonal**	**Density (calc.)**		**5.302 g/cm^3**
Space group		$R\bar{3}m$ (no. 166)	Texture parameters (March-Dollase)		axis [001] ratio 0.98967
Lattice constants, Å	*a*	3.00268(2)	2Θ range, °		6.00–115.00
	c	14.6103(2)	Step width, °		0.02
Cell volume, Å^3		114.079(2)	No. of data points		5451
Formula weight		121.38	No. of reflections calc. (α_1 only)		32
Z		3	No. of variables		46
Wavelengths, Å	α_1	1.54056	Agreement factors	$R(F^2)$	0.0411
	α_2	1.54439		R_p	0.0560
	Ratio	0.5		R_{wp}	0.0785
				χ^2	6.955

Table S2. Atomic positions in $Li_{0.8}Ni_{0.6}Sb_{0.4}O_2$.

Symbol	*Wyckoff position*	*Site symmetry*	*Atom*	*Occupancy*	*x/a*	*y/b*	*z/c*	*U_{iso}*
M1	3*a*	$\bar{3}m$	Sb<br>Ni<br>Li	0.40<br>0.5159(9)<br>0.0841(9)	0	0	0	0.0742(11)
M2	3b	$\bar{3}m$	Li<br>Ni	0.7159(9)<br>0.0841(9)	1/3	2/3	1/6	0.0187(13))
O	6*c*	3*m*		1	0	0	0.25723(13)	0.0085(5)

Table S3. Bond lengths (A) and bond angles (º) in $Li_{0.8}Ni_{0.6}Sb_{0.4}O_2$.

**Symbol**	**M1**	**M2**	**M1–O–M1**	**93.60(6) ×3**
Occupancy	$Sb_{0.40}Ni_{0.516}Li_{0.084}$	$Li_{0.716}Ni_{0.084}$	O–M1–O	93.60(6) ×6
Average radius [4]	0.80	0.89		86.40(6) ×6
M-O distance	2.0595(10)×6	2.1809(12)×6	O–M2–O	92.99(6) ×6
Radii sum [4]	2.06	2.15		87.01(6) ×6

References

1. T. Salikhov, E. Klysheva, E. Zvereva, V. Nalbandyan, I. Shukaev, B. Medvedev, E. Vavilova; Lithium diffusion in a new cathode material $Li_{0.8}[Ni_{0.6}Sb_{0.4}]O_2$ studied by ^{7}Li NMR.; Magn. Reson. Solids 18, 16207 (2016).
2. Larson, A.C., Von Dreele, R.B., General Structure Analysis System (GSAS), Los Alamos National Laboratory Report LAUR86-748 (2004).
3. Toby, B.H.; EXPGUI, a graphical user interface for GSAS; *J. Appl. Cryst.* **34**, 210-213 (2001).
4. R. D. Shannon; Revised Effective Ionic Radii and Systematic Studies of Interatomic Distances in Halides and Chalcogenides; *Acta Crystallogr.* A**32**, 751 (1976).
